# Treatment of Rubeola by Inunction

**Published:** 1850-12

**Authors:** 


*Treatment of Rubeola by Inunction.*—By John Evans, M. D., Prof. of Obstetrics, &c., in Rush Medical College, etc., etc.

June 1, 1850.—Miss F., aged 15 years, was laboring under the symptoms that characterize a violent attack of Measles. The febrile action was strong—pain in the extremities, loins and head, severe—the eyes were injected, suffused and intolerant of light—distressing nausea was constant, and the characteristic eruption was well marked upon the face, neck and breast.

I gave Dovers powder grs. viij. every six hours with free use of warm teas.

Finding no abatement of the distressing symptoms the next morning, I determined to use inunction, and, as practiced by Dr. Schneeman in Scarlatina, directed the patient to be rubbed with a piece of fat bacon over the entire cutaneous surface.

The relief was marked by the subsidence of all the distressing symptoms in a few hours, and the application was repeated twice the next day. No other treatment was applied except the free use of warm teas. The recovery was more rapid than I had before seen in such cases, and without any disagreeable sequel.

Two other members of the same family were treated by the inunction with the same favorable result.

I have since used the plan of treatment in a number of cases and with uniform and prompt relief.—*North Western Journal*

